# Supplementary material for: Extraction-free protocol combining proteinase K and heat inactivation for detection of SARS-CoV-2 by RT-qPCR
Source: PLoS One. 2021 Feb 26;16(2):e0247792. doi: 10.1371/journal.pone.0247792 (PMC7909620; doi:10.1371/journal.pone.0247792)
Supplement: S1 Fig — Representative colorimetric reactions run using COVID-19 Neokit Tecnoami, based on LAMP. Diagnostics is made according to final color of the solution: blue, positive; violet, negative. (PDF) [file pone.0247792.s001.pdf]

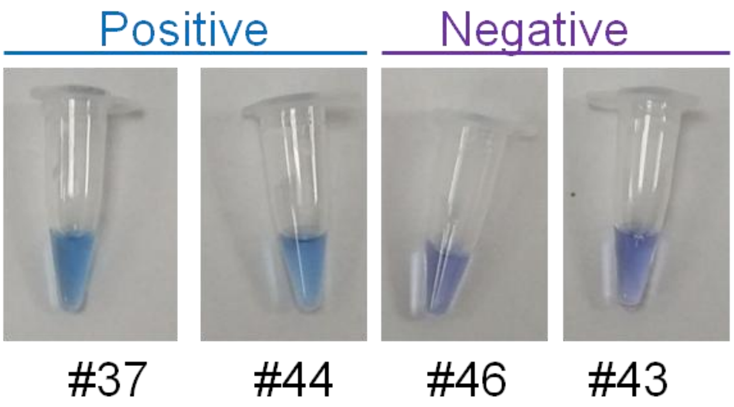

S1 Fig. PK+HID can be combined with a detection kit based on LAMP reactions. Representative colorimetric reactions run using COVID-19 Neokit Tecnoami, based on LAMP. Diagnostics is made according to final color of the solution: blue, positive; violet, negative.
