# Supplementary material for: Extraction-free protocol combining proteinase K and heat inactivation for detection of SARS-CoV-2 by RT-qPCR
Source: PLoS One. 2021 Feb 26;16(2):e0247792. doi: 10.1371/journal.pone.0247792 (PMC7909620; doi:10.1371/journal.pone.0247792)
Supplement: S2 Fig — Five positive nasopharyngeal swab samples (#1 to #5) were processed by adding 10 μl of proteinase K 10mg/ml (PK+HID samples) or 10 μl in proteinase K buffer (HID’ samples) and subjected to thermal incubations (55°C for 15 min and 98°C for 5 min). The viral N1 and N2 genes and the human RNase P gene (RP) were amplified and detected by RT-qPCR. (a) CT values obtained from RT-qPCR analysis of the same samples prepared by both different methods. (b) Ratio between relative amplicon amounts (n) of PK+HID and HID’ samples. The median of each measurement is represented with a line in the bars and the lengths of these bars represent the standard error. (c) Amplification efficiencies (EPCR). The median of each measurement is represented with a line in the bars and the lengths of these bars represent the standard error. (PDF) [file pone.0247792.s002.pdf]

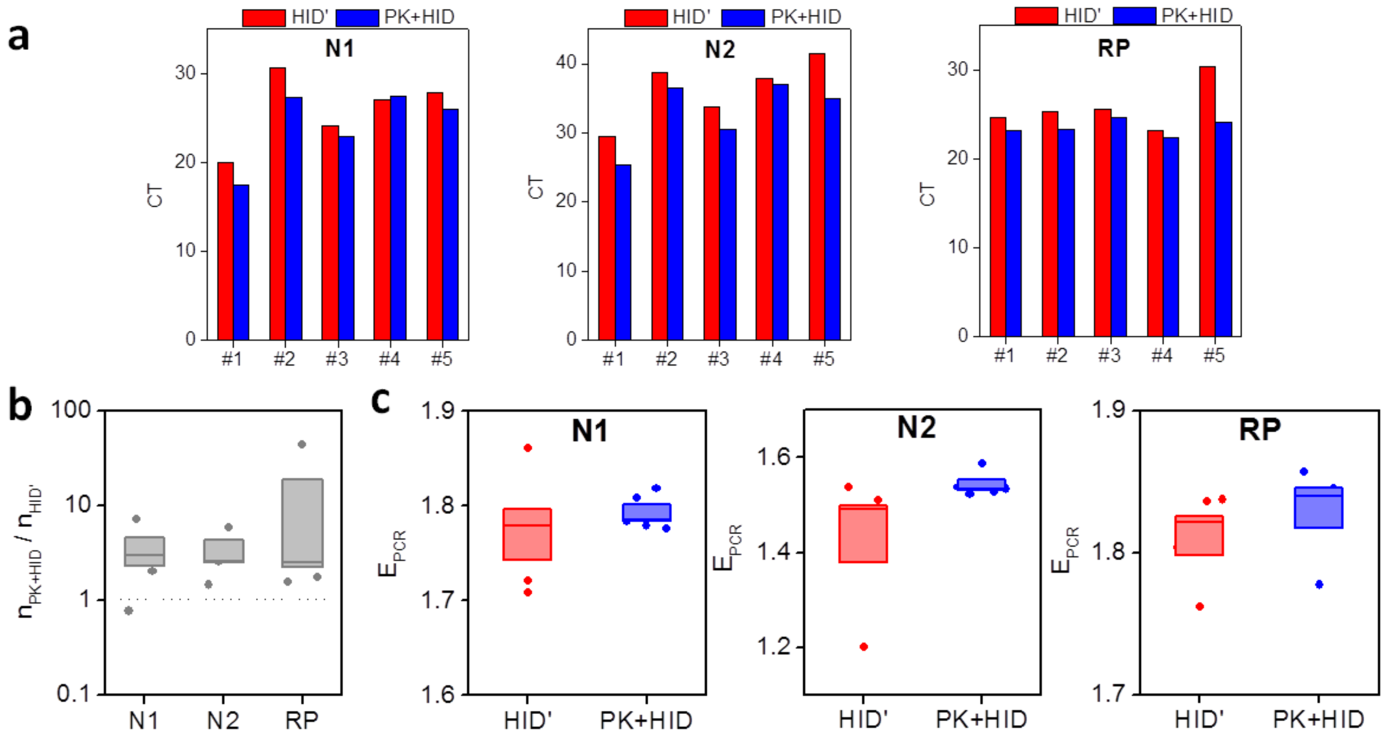

S2 Fig. Effect of saline solution dilution in PK+HID method. Five positive nasopharyngeal swab samples (#1 to #5) were processed by adding 10 µl of proteinase K 10mg/ml (PK+HID samples) or 10 µl in proteinase K buffer (HID' samples) and subjected to thermal incubations (55°C for 15 min and 98°C for 5 min). The viral N1 and N2 genes and the human RNase P gene (RP) were amplified and detected by RT-qPCR. **(a)** CT values obtained from RT-qPCR analysis of the same samples prepared by both different methods. **(b)** Ratio between relative amplicon amounts (n) of PK+HID and HID' samples. The median of each measurement is represented with a line in the bars and the lengths of these bars represent the standard error. **(c)** Amplification efficiencies (E_{PCR}). The median of each measurement is represented with a line in the bars and the lengths of these bars represent the standard error.
