# Supplementary material for: Extraction-free protocol combining proteinase K and heat inactivation for detection of SARS-CoV-2 by RT-qPCR
Source: PLoS One. 2021 Feb 26;16(2):e0247792. doi: 10.1371/journal.pone.0247792 (PMC7909620; doi:10.1371/journal.pone.0247792)
Supplement: S3 Fig — Twelve positive nasopharyngeal swab samples (#1 to #12) were processed by heat inactivation (HID samples), proteinase K treatment followed by heat inactivation (PK+HID samples) or RNA extraction (purified RNA samples). The viral N and ORF1ab genes and the human RNase P gene (RP) were amplified and detected by RT-qPCR. CT values obtained from RT-qPCR analysis of the same samples prepared by the three different methods. (PDF) [file pone.0247792.s003.pdf]

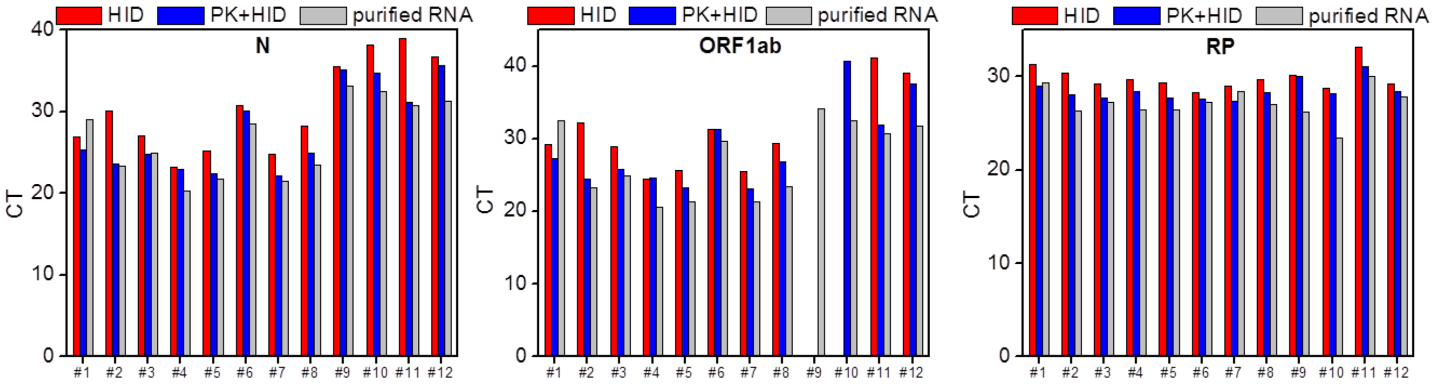

S3 Fig. Performances of HID and PK+HID methods targeting N and ORF1ab amplicons. Twelve positive nasopharyngeal swab samples (#1 to #12) were processed by heat inactivation (HID samples), proteinase K treatment followed by heat inactivation (PK+HID samples) or RNA extraction (purified RNA samples). The viral N and ORF1ab genes and the human RNase P gene (RP) were amplified and detected by RT-qPCR. CT values obtained from RT-qPCR analysis of the same samples prepared by the three different methods.
