# Supplementary material for: Extraction-free protocol combining proteinase K and heat inactivation for detection of SARS-CoV-2 by RT-qPCR
Source: PLoS One. 2021 Feb 26;16(2):e0247792. doi: 10.1371/journal.pone.0247792 (PMC7909620; doi:10.1371/journal.pone.0247792)
Supplement: S4 Fig — Ten positive nasopharyngeal swab samples were processed by PK treatment followed by heat inactivation. These PK+HID samples were kept at 4°C. The viral N and ORF1ab genes and the human RNase P gene (RP) were amplified and detected by RT-qPCR at different times of incubation at 4°C (0, 20 and 40 h). Differences between CT values obtained at different times and time = 0 h. Thin, light lines represent individual sample measurements. Strong lines represent mean + SEM values. (PDF) [file pone.0247792.s004.pdf]

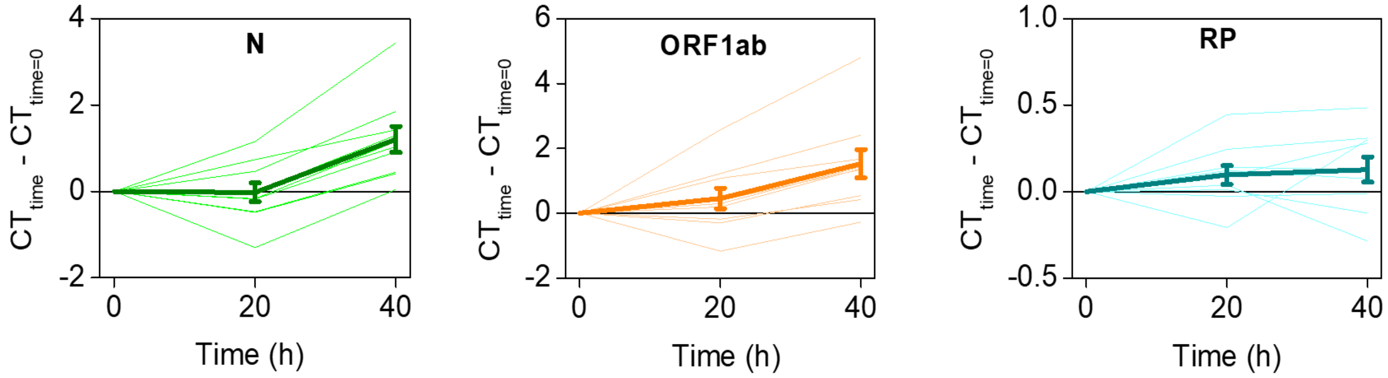

S4 Fig. Stability of PK+HID samples at 4°C. Ten positive nasopharyngeal swab samples were processed by PK treatment followed by heat inactivation. These PK+HID samples were kept at 4°C. The viral N and ORF1ab genes and the human RNase P gene (RP) were amplified and detected by RT-qPCR at different times of incubation at 4°C (0, 20 and 40 h). Differences between CT values obtained at different times and time = 0 h. Thin, light lines represent individual sample measurements. Strong lines represent mean $\pm$ SEM values.
