# Supplementary material for: Extraction-free protocol combining proteinase K and heat inactivation for detection of SARS-CoV-2 by RT-qPCR
Source: PLoS One. 2021 Feb 26;16(2):e0247792. doi: 10.1371/journal.pone.0247792 (PMC7909620; doi:10.1371/journal.pone.0247792)
Supplement: S5 Fig — Randomly-selected nasopharyngeal swab samples were simultaneously processed for RNA extraction (purified RNA) or subjected to treatment with PK 1 mg/ml (55°C for 15 min) followed by heat-inactivation at 98°C for 5 min (PK+HID). The viral N and ORF1ab genes and the human RP gene were amplified and detected by RT-qPCR. (a) CT values obtained for the same samples prepared by the two different methods; the line obtained by regression of the data (continuous lines). RP plot also includes the data obtained in SARS-CoV-2 negative samples whereas N and ORF1ab plots include those data of negative samples that provided CT values above the positive-negative cut-off. (b) Ratio between the relative amplicon copy amounts detected using PK+HID or RNA purification for those nasopharyngeal swab samples that presented CT values >30 for any of the viral genes in purified RNA samples (Ndata = 10 and 12 for N and ORF1ab genes, respectively). (PDF) [file pone.0247792.s005.pdf]

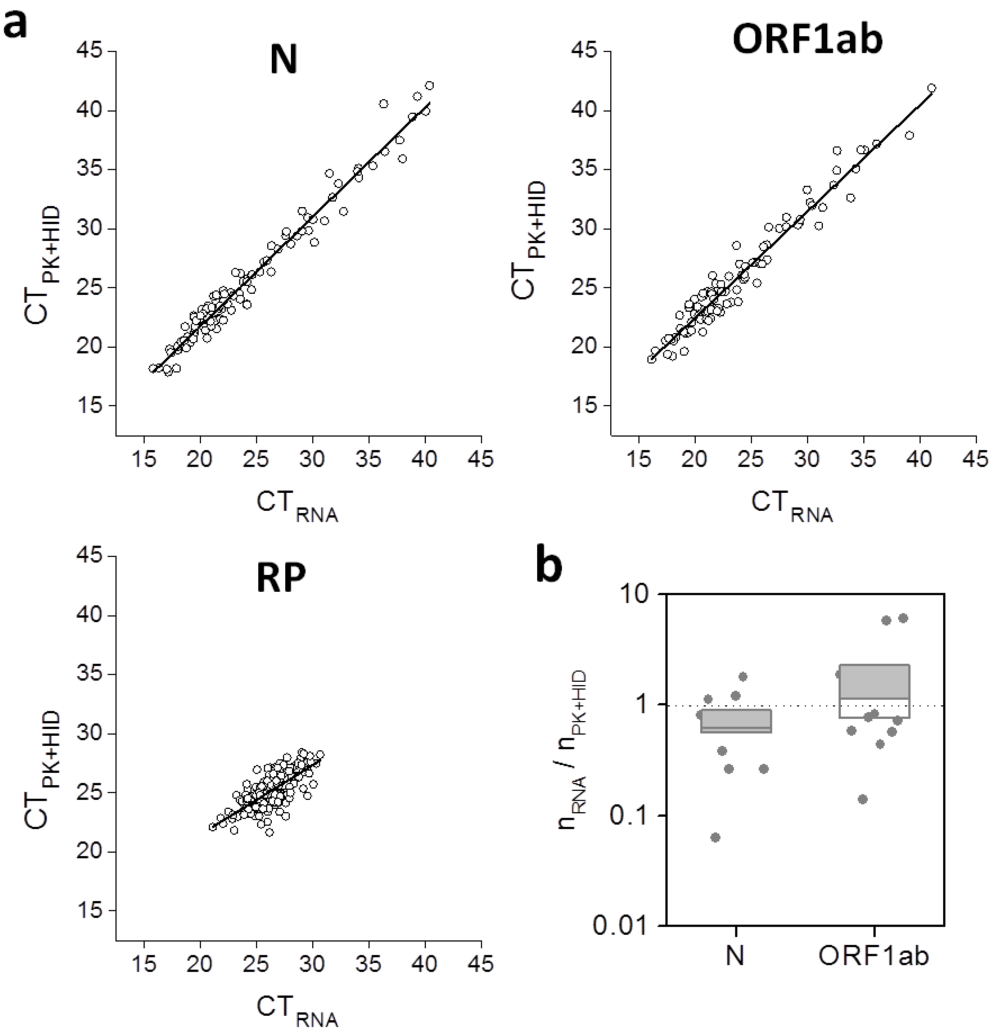

S5 Fig. Performance of PK+HID method targeting N and ORF1ab amplicons. Randomly-selected nasopharyngeal swab samples were simultaneously processed for RNA extraction (purified RNA) or subjected to treatment with PK 1 mg/ml (55°C for 15 min) followed by heat-inactivation at 98°C for 5 min (PK+HID). The viral N and ORF1ab genes and the human RP gene were amplified and detected by RT-qPCR. **(a)** CT values obtained for the same samples prepared by the two different methods; the line obtained by regression of the data (continuous lines). RP plot also includes the data obtained in SARS-CoV-2 negative samples whereas N and ORF1ab plots include those data of negative samples that provided CT values above the

positive-negative cut-off. **(b)** Ratio between the relative amplicon copy amounts detected using PK+HID or RNA purification for those nasopharyngeal swab samples that presented CT values >30 for any of the viral genes in purified RNA samples (N_{data} =10 and 12 for N and ORF1ab genes, respectively).
