# Supplementary figures and images for: Extraction-free protocol combining proteinase K and heat inactivation for detection of SARS-CoV-2 by RT-qPCR
Source: PLoS One. 2021 Feb 26;16(2):e0247792. doi: 10.1371/journal.pone.0247792 (PMC7909620; doi:10.1371/journal.pone.0247792)

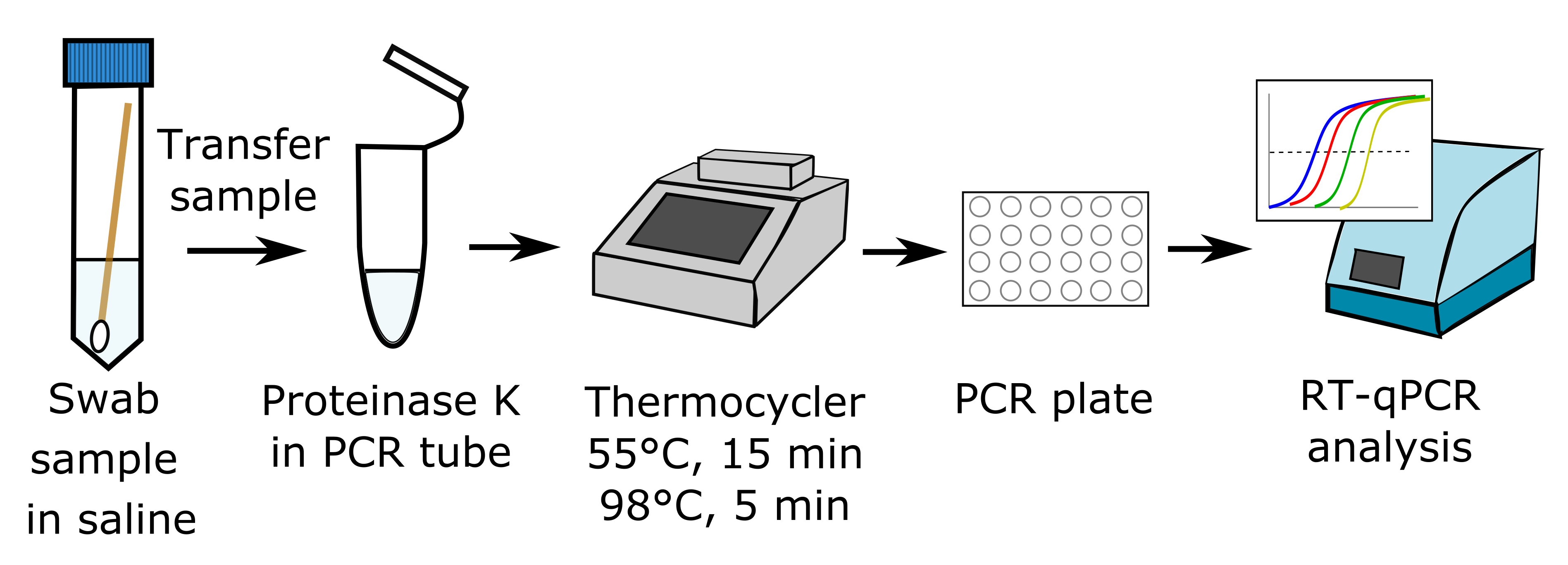

Supplement: S6 Fig — (PNG) [file pone.0247792.s006.png]
