# Supplementary material for: Extraction-free protocol combining proteinase K and heat inactivation for detection of SARS-CoV-2 by RT-qPCR
Source: PLoS One. 2021 Feb 26;16(2):e0247792. doi: 10.1371/journal.pone.0247792 (PMC7909620; doi:10.1371/journal.pone.0247792)
Supplement: S1 Table — Values are expressed as mean ± standard error. (PDF) [file pone.0247792.s007.pdf]

Amplicon	CT_{PK+HID} - CT_{RNA}	CT_{HID} - CT_{RNA}
N1	2.7 ± 0.6	5 ± 1
N2	5.6 ± 0.4	9.5 ± 0.8
RP	2.3 ± 0.5	5.5 ± 0.7

S1 Table. Mean variations in CT values in HID and PK+HID samples compared to purified RNA samples. Values are expressed as mean ± standard error.
