# Supplementary material for: Extraction-free protocol combining proteinase K and heat inactivation for detection of SARS-CoV-2 by RT-qPCR
Source: PLoS One. 2021 Feb 26;16(2):e0247792. doi: 10.1371/journal.pone.0247792 (PMC7909620; doi:10.1371/journal.pone.0247792)
Supplement: S2 Table — (PDF) [file pone.0247792.s008.pdf]

| **CT $_{5μL}$ (gene)** | **CT$_{10μL}$ (gene)** |
|---|---|
| 40.4 (N1)<br><br>23.9 (RP) | 34.0 (N1)<br><br>23.9 (RP) |
| 22.8 (N1)<br><br>22.1(RP) | 21.3 (N1)<br><br>21.4(RP) |
| 22.6 (N1)<br><br>28.6 (RP) | 21.9 (N1)<br><br>28.3 (RP) |
| 24.8 (N1)<br><br>28.4 (RP) | 24.6 (N1)<br><br>28.1 (RP) |
| 26.1 (N1)<br><br>28.9 (RP) | 25.0 (N1)<br><br>28.5 (RP) |
| 31.2 (N1)<br><br>28.6 (RP) | 30.9 (N1)<br><br>28.0(RP) |
| 29.6 (N1)<br><br>30.0 (RP) | 28.9 (N1)<br><br>29.5 (RP) |
| 34.1 (N1)<br><br>29.5 (RP) | 35.6 (N1)<br><br>29.0 (RP) |

**S2 Table.** CT values obtained in RT-qPCR analysis of the same HID+PK samples introducing 5 or 10 µl of sample volume as input for RT-qPCR.
