# Supplementary material for: Extraction-free protocol combining proteinase K and heat inactivation for detection of SARS-CoV-2 by RT-qPCR
Source: PLoS One. 2021 Feb 26;16(2):e0247792. doi: 10.1371/journal.pone.0247792 (PMC7909620; doi:10.1371/journal.pone.0247792)
Supplement: S3 Table — (PDF) [file pone.0247792.s009.pdf]

CT_{RNA} **(gene, kit)**	CT_{PK+HID} **(gene, kit)**
30.7 (N, DisCoVery)<br>30.9 (ORF1ab, DisCoVery)<br>27.4 (RP, DisCoVery)	nd (N, DisCoVery)<br>nd (ORF1ab, DisCoVery)<br>23.2 (RP, DisCoVery)
23.5 (N, DisCoVery)<br>21.1 (ORF1ab, DisCoVery)<br>26.2 (RP, DisCoVery)	nd (N, DisCoVery)<br>nd (ORF1ab, DisCoVery)<br>26.0 (RP, DisCoVery)
31.3 (N, GeneFinder)<br>31.3 (RdRp, GeneFinder)<br>32.1 (E, GeneFinder)<br>27.3 (RP, Genefinder)	40.1 (N, GeneFinder)<br>nd (RdRp, GeneFinder)<br>nd (E, GeneFinder)<br>30.3 (RP, Genefinder)
34.6 (N, GeneFinder)<br>34.5 (RdRp, GeneFinder)<br>38.8 (E, GeneFinder)<br>27.2 (RP, Genefinder)	41.3 (N, GeneFinder)<br>nd (RdRp, GeneFinder)<br>nd (E, GeneFinder)<br>28.2 (RP, Genefinder)
35.9 (N, GeneFinder)<br>37.0 (RdRp, GeneFinder)<br>nd (E, GeneFinder)<br>28.4 (RP, Genefinder)	nd (N, GeneFinder)<br>nd (RdRp, GeneFinder)<br>nd (E, GeneFinder)<br>30.6 (RP, Genefinder)

S3 Table. CT values of false-negative samples detected with the PK+HID method, using the results obtained with purified RNA samples as reference.
