# Supplementary material for: Extraction-free protocol combining proteinase K and heat inactivation for detection of SARS-CoV-2 by RT-qPCR
Source: PLoS One. 2021 Feb 26;16(2):e0247792. doi: 10.1371/journal.pone.0247792 (PMC7909620; doi:10.1371/journal.pone.0247792)
Supplement: S4 Table — (PDF) [file pone.0247792.s010.pdf]

CT values (RNA, RT-qPCR)	Diagnostic (RNA, LAMP)	Diagnostic (PK+HID, LAMP)
N: 25<br>ORF1ab: 24	positive	positive
N: 20<br>ORF1ab: 20	positive	positive
N: 28<br>ORF1ab: 27	positive	positive
N: 27<br>ORF1ab: 27	positive	positive
N: 32<br>ORF1ab: 33	positive	positive
N: 19<br>ORF1ab: 19	positive	positive
N: 32<br>ORF1ab: 31	positive	positive
N: 34<br>ORF1ab: 35	negative	negative
N: 22<br>ORF1ab: 22	negative	positive
N: 16<br>ORF1ab: 17	positive	positive
N: 25<br>ORF1ab: 26	negative	negative
N: 25<br>ORF1ab: 25	negative	positive
N: 27<br>ORF1ab: 28	negative	negative
N: 15<br>ORF1ab: 15	positive	positive
N: 28<br>ORF1ab: 29	negative	negative
N: 22<br>ORF1ab: 24	negative	negative

N: 19<br>ORF1ab: 20	positive	positive
N: 17<br>ORF1ab: 19	positive	positive
N: 26<br>ORF1ab: 27	negative	negative
N: 22<br>ORF1ab: 23	positive	negative
N: 16<br>ORF1ab: 18	positive	positive
N: 18<br>ORF1ab: 19	positive	positive
N: 21<br>ORF1ab: 21	positive	positive
N: 23<br>ORF1ab: 24	negative	negative
N: 23<br>ORF1ab: 25	positive	negative
N: 20<br>ORF1ab: 20	positive	positive
N: 18<br>ORF1ab: 19	positive	positive
N: 17<br>ORF1ab: 18	positive	positive
N: 34<br>ORF1ab: 35	negative	negative
N: 15<br>ORF1ab: 16	positive	positive
N: 34<br>ORF1ab: 35	negative	negative
N: 20<br>ORF1ab: 21	positive	positive
N: 15<br>ORF1ab: 16	positive	positive
N: 16<br>ORF1ab: 17	positive	positive
N: 19<br>ORF1ab: 20	positive	positive

N: 19<br>ORF1ab: 20	positive	positive
N: 30<br>ORF1ab: 31	negative	negative
N: 35<br>ORF1ab: 37	negative	negative
N: 32<br>ORF1ab: 33	negative	negative
N: 23<br>ORF1ab: 25	negative	negative
N: 35<br>ORF1ab: 36	negative	negative
N: 27<br>ORF1ab: 28	negative	negative

S4 Table. CT values of the positive samples processed by RNA extraction and analyzed by RT-qPCR targeting N and ORF1ab viral genes, and diagnostic results obtained with the same samples processed by RNA extraction or PK+HID and analyzed using a LAMP detection kit.
